# Clinical and epidemiological characteristics of imported dengue fever among inbound passengers: Infrared thermometer–based active surveillance at an international airport

**DOI:** 10.1371/journal.pone.0225840

**Published:** 2019-12-05

**Authors:** Chia-ping Su, Ying-Yun Wang, Kuei-Chu Ku, Chi-Tai Fang

**Affiliations:** 1 Taiwan Centers for Disease Control, Ministry of Health and Welfare, Taipei City, Taiwan; 2 Institute of Epidemiology and Preventive Medicine, College of Public Health, National Taiwan University, Taipei, Taiwan; 3 Graduate Institute of Clinical Medical Sciences, Chang Gung University, Taoyuan City, Taiwan; 4 Division of Infectious Diseases, Department of Internal Medicine, National Taiwan University Hospital, Taipei, Taiwan; The University of Hong Kong, CHINA

## Abstract

**Background:**

Dengue fever is endemic in tropical and subtropical areas, especially Southeast Asia. International air travel facilitates the spread of dengue across and within borders. To date, no predictive factors have been established for assessing risk of dengue among febrile travelers.

**Methods:**

Since 2006, Taiwan has operated a program of infrared thermometer–based non-contact active surveillance at Taoyuan International Airport (TPE). All inbound passengers from dengue-endemic countries who are febrile (tympanic temperature ≥38°C) undergo routine laboratory testing for dengue. We analyzed clinical and epidemiological characteristics of all tested passengers entering Taiwan via TPE in 2011 to identify the predictive factors of dengue infection.

**Results:**

In 2011, of the 3,719 febrile passengers from dengue-endemic countries, 74 (2.0%) had laboratory-confirmed dengue infection. Multivariable logistic regression analysis revealed that those who were aged ≥60 years (adjusted odds ratio [aOR], 8.7; 95% confidence interval [CI], 2.6–29.6) and had self-reported fever (aOR, 2.5; 95% CI, 1.5–4.1), skin rashes (aOR, 11.0; 95% CI, 3.4–35.1), or a tympanic temperature ≥39°C (aOR, 2.9; 95% CI, 1.7–4.9) were significantly more likely to have dengue (all *p* values < 0.05). Compared with travelers who stayed in dengue-endemic countries for ≤7 days, those who traveled 8–14, 15–21, 22–28, and ≥29 days were also more likely to be infected (aORs of 10.2, 14.9, 39.0 and 12.0, respectively).

**Conclusion:**

These clinical and epidemiological features can facilitate timely recognition and diagnosis of imported dengue in febrile inbound passengers and therefore help prevent domestic transmission of dengue virus.

## Introduction

Dengue fever is one of the leading arthropod-borne viral infections, with 3.2 million cases reported to the World Health Organization in 2015 [1]. The clinical severity ranges from mild and self-limited to life-threatening illness that could be fatal unless promptly recognized and appropriately treated [2]. Traditionally, the disease was confined in tropic and subtropical regions, especially Southeast Asia and Latin America [1–3]. However, advances in modern air transportation allow the spread of dengue fever to non-endemic countries via infected passengers [4, 5].

Travel-related, imported dengue fever can cause regional outbreaks in previously non-endemic regions. For example, a dengue outbreak involving more than 2,000 patients occurred during 2012–2013 in Madeira, Portugal, driven by the introduction of people infected with the virus [6, 7]. Imported dengue has also caused local outbreaks in the United States and Japan [8, 9]. Imported dengue fever cases frequently caused disastrous epidemics with thousands of patients in Taiwan [10, 11]. Previous studies in Taiwan have revealed that a systematic approach to identifying infected passengers could minimize the risk of local transmissions of virus and outbreaks [12, 13]. However, because of the high volume of travelers who have visited dengue-endemic countries or have symptoms similar to other febrile illnesses, surveillance is challenging.

Since 2003, Taiwan has used an infrared thermometer–based screening system that allows rapid, non-intrusive, non-contact surveillance for febrile passengers arriving at all international airports [14]. Febrile travelers from dengue-endemic countries have been routinely tested for dengue virus infection since 2006 [14]. Given the large number of passengers from dengue-endemic countries and the significant resources used for blood testing, the present study aimed to determine whether clinical and epidemiological information can be used to differentiate imported dengue fever from other causes of febrile illness in surveillance of travelers with infrared thermometers at airports.

## Methods

The study was conducted at the quarantine station of Taoyuan International Airport (TPE), which is the largest airport in Taiwan. Before being allowed to enter the border, inbound passengers must self-report any illness to quarantine officers. In the arrival area they pass the quarantine station, which is equipped with non-contact infrared thermometer cameras (ThermaCAM P20; FLIR, Wilsonville, OR, USA). The quarantine officers calibrate these infrared thermal cameras every day. When passengers walk by, a visual alarm alerts quarantine officers if a camera detects a surface temperature exceeding 36°C. Suspected febrile passengers are then asked to have their tympanic temperature measured. Passengers with confirmed febrile illness (tympanic temperature ≥38°C) complete a standard questionnaire that collects information on demographics, nationality, travel history before entering Taiwan, personal medical history, symptoms/signs, and a contact address and phone number in Taiwan. All information is entered in the database of the National Quarantine Surveillance System of the Taiwan Centers for Disease Control (TCDC). Because this study was for public health surveillance, it was exempt from human subject review and did not require informed consent.

For febrile passengers coming from dengue-endemic countries, quarantine officers collect blood specimens to enable testing for dengue infection. All serum samples are sent to the central laboratory of TCDC for analysis by nonstructural protein 1 (NS1) antigen rapid test, envelope membrane–specific capture immunoglobulin (Ig) M and IgG enzyme-linked immunosorbent assay (ELISA), and reverse-transcription polymerase chain reaction (RT-PCR). A diagnosis of dengue virus infection was confirmed by the finding of at least one of these criteria: NS1 antigen rapid test positivity; recent dengue infection, as indicated by ELISA results (a seroconversion in IgM antibody or a fourfold or greater change in IgG antibody titers in paired serum samples); and RT-PCR positivity for one of four serotypes of dengue viruses.

For this study, we first abstracted from the system the numbers of symptomatic passengers and passengers who were tested for dengue infection, to calculate the proportion of dengue-positive cases among tested passengers during 2007–2012. The 2009 H1N1 influenza pandemic affected passengers during 2009–2010. After further considering the data completeness and quality, we included all inbound passengers at TPE who were tested for dengue infection in 2011 and excluded those having indeterminate test results. We also abstracted the number of passengers who tested positive and negative for dengue infection in 2011 and calculated the proportion of dengue-positive cases among tested passengers by month.

To analyze the predictors of confirmed dengue virus infection among febrile passengers, we collected several key demographic and travel characteristics such as sex, age, purpose of travel, departure country, length of stay in dengue-endemic countries, and self-reported occurrence of mosquito bite within 2 weeks before entry. We asked each symptomatic passenger about having fever/chills, headache, myalgia/bone pain, fatigue, skin rashes, and any of the following upper respiratory symptoms: cough, rhinorrhea, and nasal obstruction. We categorized passengers’ ages in four groups: 0–19 years, 20–39 years, 40–59 years, and ≥60 years. We classified the purposes of travel in five categories: group tourism, independent tourism, visiting friends and relatives, business, and other. We classified the lengths of stays in dengue-endemic countries in five categories: ≤7 days, 8–14 days, 15–21 days, 22–28 days, and ≥29 days. We categorized tympanic temperatures in two groups: ≤38.9°C and ≥39.0°C.

We used a chi-square test to analyze the difference in variables between febrile passengers who tested positive and negative for dengue virus infection. We conducted univariate logistic regression analysis to generate crude odds ratios (ORs) of each variable with a *p* value <0.10. We further analyzed all these variables with statistical significance by stepwise logistic regression to predict a model of factors associated with dengue virus infection among febrile inbound passengers and generate adjusted odds ratios (aORs). Using stepwise selection, a significance level of 0.10 is required to allow a variable into the model, and a significance level of 0.05 is required for a variable to stay in the model. All tests were two-sided, and *p* values <0.05 were considered statistically significant. Data analysis was performed with SAS version 9.4 (SAS Institute, Inc., Cary, NC, USA).

## Results

**Fig 1:**
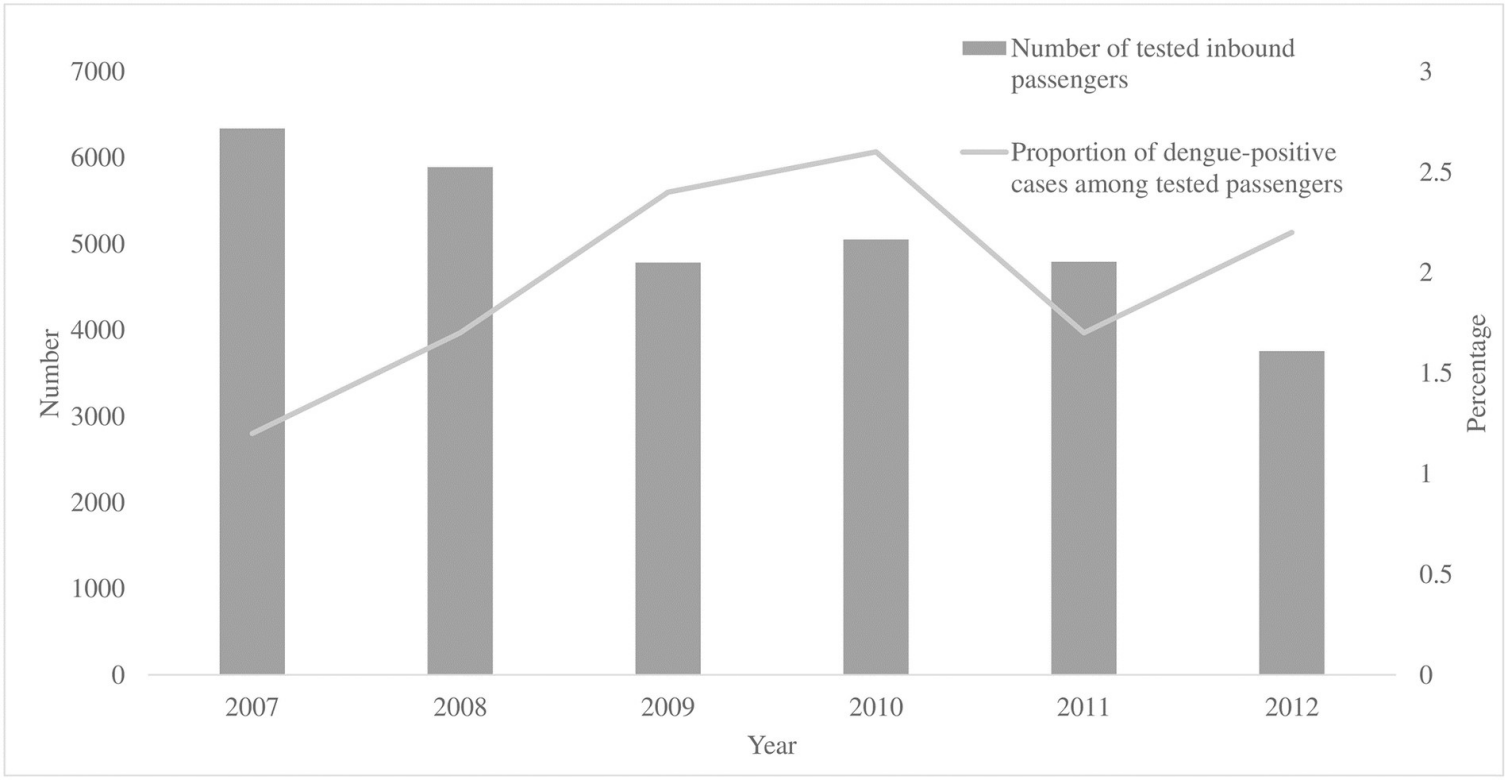
Number of tested inbound passengers and proportion of dengue-positive cases among tested passengers at Taoyuan International Airport, by year—Taiwan, 2007–2012.

**Fig 2:**
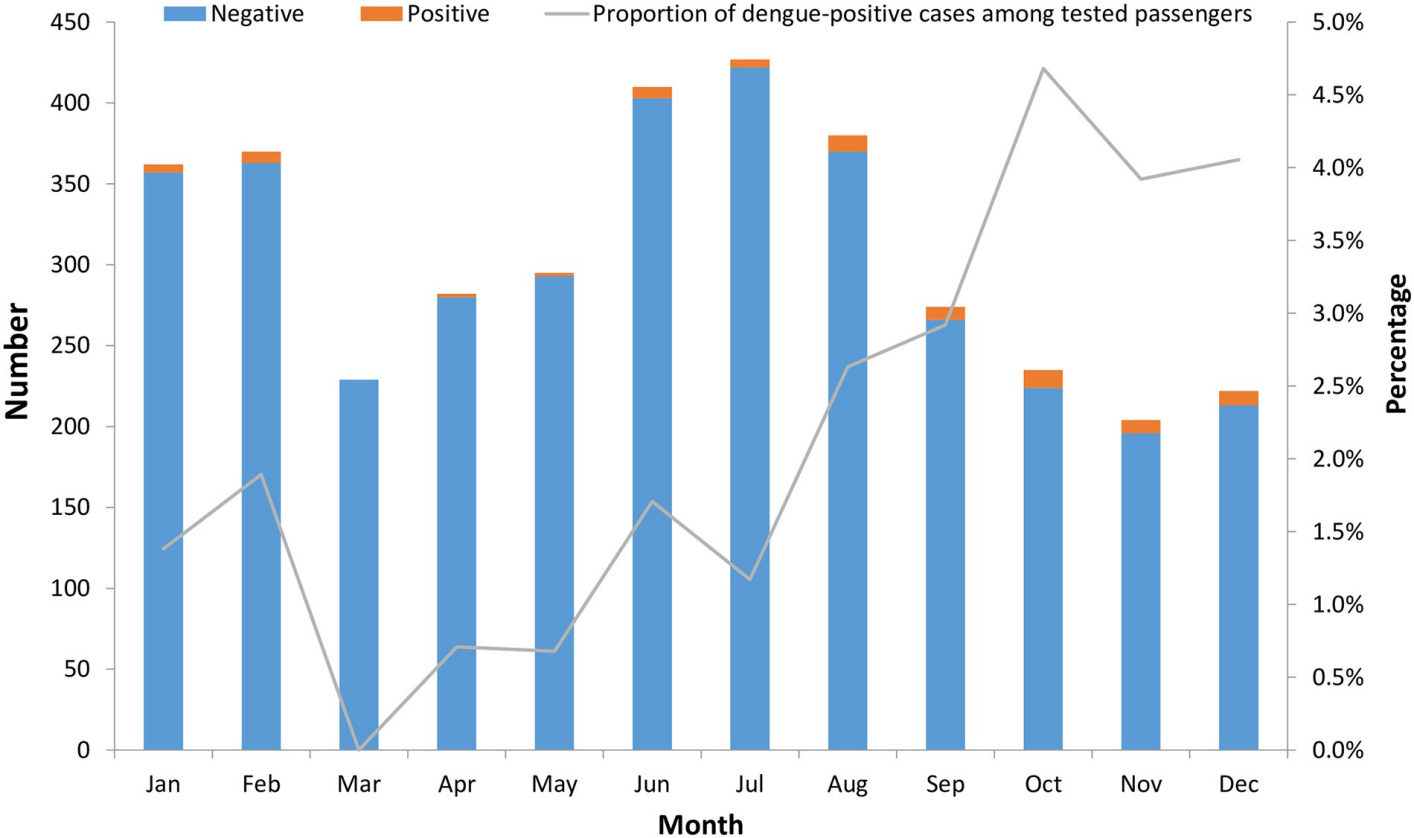
Number of tested inbound passengers and proportion of dengue-positive cases among tested passengers at Taoyuan International Airport, by results and month—Taiwan, 2011.

During 2007–2012, quarantine officers identified and tested approximately 30,000 symptomatic passengers for dengue infection. Of these, 583 (1.9%) were positive (Fig 1). Meanwhile, a total of 1,280 imported dengue cases were reported in the National Notifiable Diseases Surveillance System. In other words, 46% of the imported dengue cases in Taiwan were recognized at the airport border. In 2011, there were 11,663,768 inbound passengers at TPE, and 11,324 (0.1%) of them were identified as febrile by quarantine officers. After initial evaluation, 3,719 were tested for dengue infection, and 74 (2%) were positive. The proportion of dengue-positive cases among tested passengers largely varied by month, ranging between 0 and 4.7%, and peaked in October (Fig 2).

Among the 74 inbound passengers with laboratory-diagnosed dengue infections, 38 (51%) were positive by NS1 antigen rapid test and PCR; 5 (7%) were positive by NS1 but negative by PCR; and 23 (31%) were negative by NS1 but positive by PCR (Table 1). Of the 18 (24%) patients who were positive for recent dengue infection by ELISA, only one patient was diagnosed by a fourfold increase in IgG. The median age of patients was 32.5 years (range, 1–72 years), and 44 (59%) of the patients were male. Among the 74 patients, 21 (28%) went to visit family and relatives, 12 (16%) were on business, 11 (15%) were independent travelers, and 2 (3%) were group travelers. For the departure country, 16 patients (22%) reported traveling from Philippines, 14 (19%) from Indonesia, 10 (14%) from Thailand, 8 (11%) from Malaysia, and 6 (8%) from Vietnam. The majority of dengue patients had stayed in dengue-endemic countries for ≥29 days (58%), followed by 8–14 days (14%), 22–28 days (11%), ≤7 days (9%), and 15–21 days (7%).

**Table 1:** Number and percentage of suspected dengue cases (N = 3,690) among inbound passengers at Taoyuan International Airport, by test results and selected characteristics—Taiwan, 2011.

Characteristic	No. (%) positive for dengue, *n* = 74	No. (%) negative for dengue, *n* = 3,616	*P* value
**Sex**			
Male	44 (59.5)	1,798 (49.7)	0.10
Female	30 (40.5)	1,818 (50.3)	
**Laboratory result by NS1 and PCR**			
NS1 positive, PCR positive*	38 (51.4)	-	
NS1 positive, PCR negative	5 (6.8)	-	
NS1 negative, PCR positive*	23 (31.1)	-	
NS1 negative, PCR negative	8 (10.8)		
**Laboratory result by ELISA**			
ELISA confirmed recent infection	18 (24.3)		
**Age, years**			
0–19	5 (6.8)	632 (17.5)	0.03
20–39	44 (59.5)	2,108 (58.3)	
40–59	18 (24.3)	702 (19.4)	
≥60	7 (9.5)	174 (4.8)	
**Purpose of travel**			
Group travel	2 (2.7)	1,444 (39.9)	<0.01
Independent travel	11 (14.9)	530 (14.7)	
Visiting friends or relatives	21 (28.4)	531 (14.7)	
Business	12 (16.2)	284 (7.8)	
Other^†^	28 (37.8)	827 (22.9)	
**Departure country**			
Thailand	10 (13.5)	907 (25.1)	0.10
Indonesia	14 (18.9)	685 (18.9)	
Philippines	16 (21.6)	515 (14.2)	
Vietnam	6 (8.1)	416 (11.5)	
Malaysia	8 (10.8)	382 (10.6)	
Other	20 (27.0)	711 (19.7)	
**Stay in dengue-endemic countries, days**			
≤7	7 (9.5)	1,905 (52.7)	<0.01
8–14	10 (13.5)	248 (6.9)	
15–21	5 (6.8)	101 (2.8)	
22–28	8 (10.8)	59 (1.6)	
≥29	43 (58.1)	1,061 (29.3)	
**Fever/chills**			
Yes	35 (47.3)	1,026 (28.4)	<0.01
**Headache**			
Yes	10 (13.5)	348 (9.6)	0.26
**Myalgia/bone pain**			
Yes	7 (9.5)	202 (5.6)	0.15
**Fatigue**			
Yes	3 (4.1)	51 (1.4)	0.06
**Skin rashes**			
Yes	5 (6.8)	15 (0.4)	<0.01
**Upper respiratory symptoms**			
Yes	5 (6.8)	622 (17.2)	0.02
**Tympanic temperature (°C)**			
≤38.9	50 (67.6)	3,074 (85.0)	<0.01
≥39.0	24 (32.4)	542 (15.0)	
**Mosquito bite 2 weeks before entry**			
Yes	12 (16.2)	441 (12.2)	0.31
No	61 (82.4)	3,100 (85.7)	

*PCR results by virus type: Type 1, 18 cases (29.5%); Type 2, 20 cases (32.8%); Type 3, 13 cases (21.3%); Type 4, 10 cases (16.4%).

^†^Includes visiting from other countries for sightseeing or working in Taiwan.

The most commonly reported symptoms among dengue patients were fever/chills (35 patients, 47%), followed by headache (10 patients, 14%), myalgia/bone pain (7 patients, 9%), skin rashes (5 patients, 7%), and fatigue (3 patients, 4%). (Table 1). Results of univariate analysis revealed statistically significant association of dengue virus infection with passenger age, purpose of travel, length of stay in dengue-endemic countries, and self-reported fever, fatigue, skin rashes, and tympanic temperature ≥39.0°C (Table 2). Stepwise logistic regression analysis indicated that older age was more associated with dengue virus infection than was younger age. The odds of being dengue-positive for those aged 20–39, 40–59, and ≥60 years were 3.2, 5.4, and 8.7-fold higher than those aged 0–19 years, respectively.

**Table 2:** Univariate and multivariable analyses of factors associated with dengue infection among inbound passengers at Taoyuan International Airport—Taiwan, 2011.

	Univariate Analysis		Multivariable Analysis	
Variable	Odds ratio (95% CI)	*P* value	Adjusted odds ratio (95% CI)	*P* value
**Age, years**				
0–19	Reference		Reference	
20–39	2.6 (1.0–6.7)	0.04	3.2 (1.2–8.3)	0.02
40–59	3.2 (1.2–8.8)	0.02	5.4 (1.9–15.2)	<0.01
≥60	5.1 (1.6–16.2)	<0.01	8.7 (2.6–29.6)	<0.01
**Purpose of travel**				
Independent travel	Reference		-	-
Group travel	0.04 (0.01–0.2)	<0.01	-	-
Visiting friends or relatives	0.5 (0.3–1.1)	0.09	-	-
Business	1.1 (0.5–2.2)	0.86	-	-
Other	0.9 (0.5–1.5)	0.60	-	-
**Stay in dengue-endemic countries, days**				
≤7	Reference		Reference	
8–14	10.8 (4.1–28.5)	<0.01	10.2 (3.8–27.3)	<0.01
15–21	13.9 (4.5–44.7)	<0.01	14.8 (4.5–48.8)	<0.01
22–28	39.4 (13.8–112.4)	<0.01	39.0 (13.1–116.1)	<0.01
≥29	11.4 (5.1–25.4)	<0.01	12.0 (5.3–27.1)	<0.01
**Fever/chills**				
No	Reference		Reference	
Yes	2.3 (1.4–3.6)	<0.01	2.5 (1.5–4.1)	<0.01
**Fatigue**				
No	Reference		-	-
Yes	3.0 (0.9–9.7)	0.07	-	-
**Skin rashes**				
No	Reference		Reference	
Yes	17.4 (6.2–49.2)	<0.01	10.1 (3.4–35.1)	<0.01
**Upper respiratory tract symptoms**				
No	Reference		Reference	
Yes	0.4 (0.1–0.9)	0.02	0.4 (0.1–1.0)	0.05
**Tympanic temperature (°C)**				
≤38.9	Reference		Reference	
≥39.0	2.7 (1.7–4.5)	<0.01	2.9 (1.7–4.9)	<0.01

CI = Confidence interval.

Moreover, passengers who had self-reported fever (aOR, 2.5; 95% confidence interval [CI], 1.5–4.1), skin rash (aOR, 10.1; 95% CI, 3.4–35.2), and tympanic temperature ≥39°C (aOR, 2.9; 95% CI, 1.7–4.9) were significantly more likely to have dengue, whereas those with respiratory tract symptoms were not (aOR, 0.4; 95% CI, 0.1–1.0). Longer length of stay in dengue-endemic countries was associated with increased likelihood of testing positive for dengue infection. Compared with those who traveled for ≤7 days, those who traveled 8–14, 15–21, 22–28, and ≥29 days were more likely to be infected (aORs of 10.2, 14.9, 39.0 and 12.0, respectively). The proportion of the variance explained by the multivariable logistic regression model was 0.2086.

## Discussion

This study showed an association between dengue virus infection in febrile incoming passengers and certain characteristics: older age, longer stays in dengue-endemic countries, self-reported fever, skin rashes, and tympanic temperature ≥39°C. To our knowledge, this is the first study examining data from active surveillance for dengue among all inbound passengers at an international airport. Dengue has been reported as the most common cause of febrile illness among people who seek medical care after travel to Latin America or Asia [4], but the epidemiological characteristics and clinical details of dengue patients among returning travelers remain unknown. The majority of previous studies analyzing the predictors of dengue virus infection among travelers were conducted in hospital settings, thus excluding those who were infected but did not seek medical attention. The current findings may be useful to public health workers for identifying imported dengue cases among inbound travelers and to clinicians for diagnosing travelers with suspect dengue infection and providing them with adequate treatment.

Following the global outbreak of severe acute respiratory syndrome in 2003, many countries started to establish entry screening systems using non-contact infrared thermometers at international airports for detecting febrile passengers [14–19]. After publication of the International Health Regulations (2005) by the World Health Organization, exit and entry screening became mandatory in certain situations for the member states [20, 21]. However, the efficiency and effectiveness of an infrared thermometer–based fever screening system at the border for detecting imported diseases and preventing local disease transmission remains controversial [15, 22]. A previous review article concluded that the effectiveness of non-contact infrared thermometers in detecting symptomatic passengers was limited when fever prevalence among passengers was less than 1% [23]. In addition, environmental factors such as room temperature and humidity can affect the measurements [24]. Although other factors might affect the sensitivity and specificity of infrared thermometers for disease detection, the overall efficiency of screening could be increased with the addition of a questionnaire on travel history and symptoms.

Prompt recognition and isolation of inbound passengers with dengue at borders could prevent local transmission of diseases. A prospective study of adult short-term travelers from the Netherlands to dengue-endemic areas showed that seroconversion for antibodies to dengue virus occurred in 1.2% of travelers at risk [25]. Although the proportion of dengue-positive cases among tested febrile passengers was relatively low in this study, approximately half of imported dengue cases in Taiwan were identified at the airport border. Early diagnosis and recognition can prevent dengue virus transmission in the community. To improve the efficiency and decrease the overall cost of surveillance, it might be efficient to aim screening toward the specific risk groups identified in this study. This is extremely important in Taiwan because of the large number of inbound passengers from Southeast Asia, where dengue is endemic.

Rapid, convenient diagnostic testing for acute-phase dengue is crucial to early identification of imported cases among febrile passengers at borders. Although IgM and IgG ELISAs for dengue are widely used in routine laboratories, there are variations in detection limits in the acute phase of dengue [26]. In addition, serologic diagnosis of dengue may require testing of a convalescent-phase specimen, which is usually not available in this setting. NS1 antigen detection kits can be used in field settings with limited equipment, such as at quarantine stations, and can provide results in less than an hour [26]. However, the sensitivity of NS1 antigen rapid testing in this study was only approximately 60%. Detection of viral RNA by RT-PCR allows early diagnosis in the febrile phase. The combination of NS1 and PCR detected approximately 90% of dengue cases in this study and therefore should be considered for diagnosis of dengue at borders.

A previous study revealed that several major cities in the United States, Europe, Japan, and Australia, where dengue is not currently endemic, had a high risk of importing dengue-infected travelers because of their large volumes of international travelers [27]. An additional international concern is Zika virus, an emerging mosquito-borne disease with serious perinatal and neurologic complications. Outbreaks have been reported throughout the Americas and several regions of the world since 2015. Taiwan quickly adopted Zika surveillance at international airports and has detected several imported Zika cases through this system [28]. When emerging/re-emerging diseases become international public health concerns, travel and border health measures play important roles in national disease prevention strategies.

This study found that old age could be used as a predictor of dengue virus infection among inbound passengers with febrile illness. This finding is consistent with previous results showing a higher likelihood for older adults to be infected with dengue [29, 30]. Compared with those of a younger age, older persons could be more likely to develop clinical dengue with typical symptoms/signs that can be detected by this surveillance system. Furthermore, the severity of the disease also increases with the age of the patient [31], and older age has been found to be an important determinant of mortality in several studies [32, 33]. Early recognition of dengue in senior passengers can potentially improve the outcome of infection through appropriate supportive treatment.

Longer length of stay in dengue-endemic countries was also found to be associated with dengue virus infection among febrile passengers. A previous study revealed that length of stay was correlated with seropositivity [34]. The finding is not surprising, because long-term travelers are clearly at higher risk of exposure to dengue virus than short-term travelers. However, the incubation period of dengue and the time of screening should also be considered in this study setting. The incubation period of dengue is 4–10 days after the bite from an infected mosquito. Therefore, travelers who stayed in dengue-endemic countries <7 days are less likely to present at the border with fever, even if they have been infected. In addition, previous studies also showed that purpose of travel could be an important predictor of dengue infection [25, 35]. For example, group travelers tend to stay shorter and hence have a lower risk of dengue infection than travelers who stay longer for visiting friends and relatives or for business. Univariate analysis results indeed showed a lower risk of dengue infection among group travelers than other travelers. However, further stepwise regression analysis didn’t select for travel purpose in the model. Such a discrepancy could be explained by the high correlation between length of stay and the purpose of the travel. Therefore, targeting for screening the febrile passengers who did not participate in travel groups or who stayed in dengue-endemic countries for more than 1 week could increase the possibility of detecting dengue virus infection at international airports and could reduce unnecessary testing.

Travelers who plan to visit dengue-endemic countries should be advised to take preventive measures to avoid mosquito bite, such as properly using repellents, protective clothing, and insecticides [36]. This study found that a self-reported history of mosquito bite was not associated with dengue virus infection, and prior research also has confirmed it to be of little help for diagnosis of dengue [37]. Travelers with dengue virus infection might not be aware of or might not recall a mosquito bite. Therefore, even when patients deny mosquito bite, physicians should consider the possibility of dengue infection. Until an effective, safe, affordable vaccine against dengue virus becomes available on the market, travelers should be educated beforehand about the risk of mosquito-borne infection and methods of prevention to avoid travel-related infection.

Fever is a common symptom in travelers. Evaluation of fever in a returning traveler should take into account the clinical findings, the travel destination, and the incubation period of relevant possible infections [4, 38]. Typical dengue fever is an acute febrile illness accompanied by headache, skin rashes, retro-orbital pain, and marked bone pain. However, the majority of infections are asymptomatic or have non-specific symptoms [1–3]. A study reviewing dengue infection among international travelers visiting Bali, Indonesia, showed that the most common clinical manifestation was fever, followed by myalgia, headache, and malaise. Rash was found in only 9.8% of the patients [39]. This study found that dengue was associated with only fever/chills. Skin rashes could be a specific symptom for dengue, but the number of cases was relatively small. Therefore, when screening inbound passengers in a resource-limited setting, high fever may be the most important single symptom or sign for detecting dengue infection.

Upper respiratory symptoms, including cough, rhinorrhea, and sore throat, are often recognized as uncommon presentations among patients with dengue infection. However, a review of patients with laboratory-confirmed dengue in Taiwan showed that approximately 40% of patients had cough [40]. A study conducted in Vietnam revealed that the frequency of cough was similar in both dengue and non-dengue cases [41]. Even though this study found a negative association between upper respiratory symptoms and dengue virus infection, the upper limit of the 95% confidence interval was very close to 1. This association could also be influenced by the activity of other respiratory pathogens that could cause febrile illness, for example, influenza virus. Hence, in a primary care setting or at the airport border, physicians or quarantine officers should not use only upper respiratory symptoms to exclude a diagnosis of dengue in febrile travelers.

The findings in this study are subject to at least four limitations. First, active surveillance at the airports identified only the febrile inbound passengers whose fevers were detected by infrared thermometer or who reported to the quarantine station voluntarily. Some passengers might take antipyretics to avoid fever detection and testing, which would lead to underestimation of the number of dengue infections among inbound passengers. Second, although all inbound passengers from dengue-endemic countries with febrile illnesses are required to be tested for dengue virus infection, the quarantine officers made decisions about blood sampling on a case-by-case basis, so the inclusion criteria were not consistent. For example, an infant or young child with febrile illness might not undergo blood sampling because of a lack of equipment or training. Third, all information collected from the questionnaire was self-reported, including demographics, travel history before entering Taiwan, and symptoms. Despite careful review, misclassification might have occurred because of incomplete or inaccurate self-reports. Nonetheless, because the patients were asked to complete questionnaire before the diagnosis of dengue, recall bias would not have differentially affected dengue patients from non-dengue patients. Finally, the results in this study were based on data from the year 2011, and the results may differ by year. The proportion of dengue-positive cases among inbound passengers with all causes of fever also could differ by year.

In conclusion, analyzing data from non-contact infrared thermometer–based active surveillance at an international airport revealed that dengue virus infection in febrile inbound passengers was associated with older age, a stay in dengue-endemic countries exceeding 1 week, skin rashes, and higher body temperature. Targeted screening of passengers with these characteristics could increase the efficiency of active surveillance for dengue infection at the border and could help prevent virus transmission in the community. Travelers should be advised to take preventive measures against mosquito bites before visiting dengue-endemic areas.
